# Developmental expression of human tau in *Drosophila melanogaster* glial cells induces motor deficits and disrupts maintenance of PNS axonal integrity, without affecting synapse formation

**DOI:** 10.1371/journal.pone.0226380

**Published:** 2019-12-10

**Authors:** Enrico M. Scarpelli, Van Y. Trinh, Zarrin Tashnim, Jacob L. Krans, Lani C. Keller, Kenneth J. Colodner

**Affiliations:** 1 Frank H. Netter, M.D. School of Medicine, Quinnipiac University, North Haven, CT, United States of America; 2 Department of Biological Sciences, Quinnipiac University, Hamden, CT, United States of America; 3 Program in Neuroscience and Behavior, Mount Holyoke College, South Hadley, MA, United States of America; 4 Department of Neuroscience, Western New England University, Springfield, MA, United States of America; Biomedical Sciences Research Center Alexander Fleming, GREECE

## Abstract

Tauopathies are a class of neurodegenerative diseases characterized by the abnormal phosphorylation and accumulation of the microtubule-associated protein, tau, in both neuronal and glial cells. Though tau pathology in glial cells is a prominent feature of many of these disorders, the pathological contribution of these lesions to tauopathy pathogenesis remains largely unknown. Moreover, while tau pathology is predominantly found in the central nervous system, a role for tau in the cells of the peripheral nervous system has been described, though not well characterized. To investigate the effects of glial tau expression on the development and maintenance of the peripheral nervous system, we utilized a *Drosophila melanogaster* model of tauopathy that expresses human wild-type tau in glial cells during development. We found that glial tau expression during development results in larval locomotor deficits and organismal lethality at the pupal stage, without affecting larval neuromuscular junction synapse development or post-synaptic amplitude. There was, however, a significant decrease in the decay time of synaptic potentials upon repeated stimulation of the motoneuron. Behavioral abnormalities were accompanied by glial cell death, disrupted maintenance of glial-axonal integrity, and the abnormal accumulation of the presynaptic protein, Bruchpilot, in peripheral nerve axons. Together, these data demonstrate that human tau expression in *Drosophila* glial cells does not affect neuromuscular junction synapse formation during development, but is deleterious to the maintenance of glial-axonal interactions in the peripheral nervous system.

## Introduction

Tauopathies are a class of over 20 sporadic and familial neurodegenerative diseases characterized by the formation of filamentous aggregations of the microtubule-associated protein, tau[1]. Despite the presence of these inclusions in both neuronal and glial cells[2], most research has characterized the pathological consequences of tau aggregation in neurons only, with less attention focused on the potential and unique contribution of tau aggregates residing in glial cells[3]. This is significant considering that glial cells (e.g. astrocytes, oligodendrocytes, Schwann cells) play an active and essential role in the function and maintenance of the nervous system[4,5], and studies in animal glial tauopathy models have shown that the accumulation of tau in glial cells is deleterious to normal brain physiology. For example, transgenic mice that express human tau in astrocytes or oligodendrocytes display neurodegenerative changes concomitant with disease-associated glial tau pathology in aged mice[6,7]. Moreover, glial cell dysfunction is associated with the neurodegeneration and brain pathology in tauopathies[8]. While these studies support the notion that glial cells contribute to tauopathy pathogenesis, the specific contributions of glial cells to nervous system degeneration remains poorly understood.

In addition to central nervous system (CNS) pathology, emerging evidence points to a role for tau in the peripheral nervous system (PNS)[9]. Along with the major tau isoforms found in the CNS, a high-molecular weight tau isoform has been identified in the PNS[10,11], and while its function is unclear[12], tau physiology has been shown to be crucial for PNS integrity. For instance, recent studies have shown that mice deficient of all tau isoforms display motor deficits and peripheral sciatic nerve dysfunction[13]. Moreover, transgenic overexpression of tau throughout the entire nervous system results in PNS sciatic nerve aberrations and motor deficits[14], raising the question of whether tau may play a role in peripheral nervous system function. Importantly, glial cells play a critical role in the development and early maintenance of the nervous system, including the peripheral nervous system, and tau-mediated disruption of these developmental processes has the potential to impact long-term health and function. In both tau knockout and overexpression mouse models, sciatic nerve abnormalities are associated with marked myelin degeneration[13,14]. Schwann cells and oligodendrocytes, the major myelinating cells of the PNS and CNS, respectively, myelinate axons and facilitate system integration and coordination across sensory modalities. While Schwann cell-specific tau transgenic mice have not been generated, co-expression of tau in neuronal and glial cells, including Schwann cells, has been shown to result in neurodegeneration and Schwann cell death[15]. Tau is similarly toxic to myelinating cells in the CNS as oligodendrocyte-specific tau expression disrupts the maintenance of myelin integrity[7], consistent with *in vitro* experiments overexpressing tau in cultured oligodendrocytes[16]. Considering these observed consequences of tau expression in glial cells, as well as the critical contribution of glia in the development of the nervous system[17], we set out to determine whether glial tau expression disrupts the development and/or early maintenance of the PNS.

The fruit fly, *Drosophila melanogaster*, serves as a useful model for studying the developmental effects of glial tau expression given the versatile genetic tools available for manipulating cell-type specific protein expression and the accessibility of the *Drosophila* PNS. Importantly, glial cell function in nervous system development and maintenance is well-conserved between vertebrates and the specialized glial cells of the fly[18]. For example, *Drosophila* astrocytes promote synapse formation during development and regulate neurotransmitter levels at the synapse, similar to their vertebrate counterparts[18,19]. And, while *Drosophila* do not contain myelin, they do contain ensheathing glial cells that help compartmentalize the neuropil of the CNS[20]. Moreover, in the PNS, flies contain wrapping glia that encase sensory and motor segmental nerves, with glial membranes extending into the synaptic region of the neuromuscular junction (NMJ)[21,22]. Studies in *Drosophila melanogaster* glial tauopathy models have shown that glial expression of tau in adult *Drosophila* induces age-dependent degeneration[23], similar to murine models. However, to generate this adult *Drosophila* glial tauopathy, it was necessary to circumvent developmental lethality associated with early glial tau expression. That is, glial tau expression was suppressed during fruit fly development, as the embryo transitioned from larva to pupa, and was only turned on once the adult eclosed from the pupal casing. This need to prevent glial tau expression during initial developmental processes underscores the potentially significant deleterious effects of glial tau in disrupting nervous system development. Considering this glial tau-induced developmental lethality, and the importance of glial cells in nervous system development, we set out to characterize the mechanism by which glial tau expression disrupts PNS development and function in *Drosophila*.

Here, we utilized a temperature-sensitive expression system[24] to express human tau in *Drosophila* glial cells and assessed its effect on *Drosophila* PNS development and maintenance in developing *Drosophila*. More specifically, we relied on the highly stereotyped developmental program of synapse formation at the larval *Drosophila* NMJ, and the intimate contact between PNS glial cells and neuronal axons as an accessible system in which to interrogate the effect of glial tau expression on neuronal-glial interactions. This model represents a novel system in which to interrogate the pathological effects of glial tau expression on neuronal-glial dynamics and describes a useful model in which to identify mechanisms of glial tau toxicity.

## Material and methods

### *Drosophila* stocks and genetics

*tub-GAL80*^*TS*^, *w*^*1118*^, *UAS-CD8GFP*, and *UAS-CD8-PARP1-Venus* were obtained from the Bloomington Stock Center and *repo-GAL4* and *UAS-tau*^*WT*^ (0N4R) were kindly provided by Dr. Mel Feany. Double recombinant control (*repo-GAL4*,*tub-GAL80*^*TS*^) and triple recombinant tau transgenic (*repo-GAL4*,*tub-GAL80*^*TS*^,*UAS-tau*^*WT*^) flies were balanced over *TM6B*,*Tb*. Crosses were performed at 18°C or 28°C in incubators with humidity control and 12-hour light:dark cycles. Flies were maintained on standard *Drosophila* Bloomington food (Nutri-Fly, Genesee Scientific).

### Pupal development and adult eclosion rates

To determine proportion of expected pupae, *repo-GAL4*,*tub-GAL80*^*TS*^*/TM6B*,*Tb* (control) and *repo-GAL4*,*tub-GAL80*^*TS*^,*UAS-tau*^*WT*^*/TM6B*,*Tb* (tau) were crossed to isogenic *w*^*1118*^ flies to segregate the *TM6B*,*Tb* (tubby) balancer from control and tau transgenic pupae. Total number of pupae with and without the tubby phenotype was determined from at least three independent experiments. To determine the number of adult flies to eclose, adult flies with and without the humoral bristle marker (*TM6b*) were counted from at least three independent experiments.

### Larval motility assay

Third instar crawling larvae were placed on an agar grape plate that had been warmed to the appropriate temperature, and the number of body wall contractions per minute was manually quantified under a dissecting microscope at room temperature. One body wall contraction was defined as a full completion of both phases of a periodic larval stride: the initial forward movement of the head, tail and gut, followed by the movement of the body wall wave of each abdominal segment in the direction of the movement [25]. Full contractions that did not result in actual forward movement were included. Representative images and larval crawling videos were recorded at room temperature using either Moticam 2.0 software connected to a Moticam 3.0MP digital camera or EverFocus Polestar II digital camera connected to a Blackline DVR.

*Video analysis*: Videos of larval crawling were analyzed in Ethovision XT15 (Noldus Information Technology). Following a 15 second acclimation period to the plate, total distance travelled, mean velocity, and maximum acceleration were assessed for a two minute period using dynamic subtraction thresholding, and successful identification of the larvae were verified by the experimenter. Data for larval locomotor parameters were exported and analyzed in GraphPad Prism.

### Immunofluorescence staining

Wandering third-instar larvae were dissected and stained according to standard procedures[26]. Primary antibodies were used at the following dilutions: BRP (nc82, 1:100; Developmental Studies Hybridoma Bank, AB_2314866, mouse monoclonal); GFP (1:1000, Millipore-Sigma, AB3080, rabbit polyclonal); AT8 (1:1000; ThermoFisher Scientific, MN1020, mouse monoclonal); cleaved PARP (1:1000; Cell Signaling, 9541, rabbit polyclonal). Secondary Alexa Fluor antibodies (goat anti-mouse 488, goat anti-rabbit 555 or 594, and goat anti-rabbit 647) and Cy3- and Cy5-conjugated HRP were obtained from Jackson ImmunoResearch Laboratories Inc. and used at a 1:300 dilution. To visualize DNA, Hoechst (1:1000; Abcam) was added during the secondary antibody incubation step or mounting media with DAPI was used (Fluoroshield, Sigma).

### Imaging and analysis

Images were digitally captured using either EZ-C1 Nikon software on a Nikon D-Eclipse C1 or NIS-Elements C software on a Nikon C2 laser scanning confocal microscope and analyzed using Fiji software. Z-stacks were combined into a single maximum projection image and axonal BRP immunofluorescence was quantified as previously described[27]. Bouton number was determined by counting the total number of boutons on muscle 6/7, independent of muscle area.

### Quantitative PCR (qPCR)

Five third instar larvae per biological replicate were homogenized in Trizol (Fisher Scientific) and RNA was extracted according to standard protocols[28]. RNA concentrations and purity were measured with a NanoDrop ND-1000 Spectrophotometer. 250 ng of RNA was reverse transcribed using SuperScript First Strand Synthesis Kit (Invitrogen), followed by qPCR using SYBR Green (Applied Biosystems). Each condition represents the data from five biological replicates, each composed of three technical replicates. *RpL32* was used as the internal control gene and primer efficiency for both primer pairs was determined to be between 94–100%. Primers used were: *Brp*: (Forward) CTA CCG ATG CCG ATA CCA ATA C; (Reverse) GTG TGT GTC CCT GTG TTT ATC T and *RpL32*: (Forward) CCA GTC GGA TCG ATA TGC TAA G; (Reverse) CCG ATG TTG GGC ATC AGA TA.

### Electrophysiological recordings

Wandering, third-instar larvae were collected from the sides of their culture vials and then placed immediately onto a dissecting dish containing a modified hemolymph-like (HL3.1) *Drosophila* saline (pH 7.15)[29]. Intracellular recordings were obtained with sharp microelectrodes, pulled from thin-wall monofilament glass (WPI) using a Flaming-Brown microelectrode puller (P-97, Sutter Instrument), and filled with 3M KCl. Recordings of compound EJPs were made from longitudinal body wall muscles 6/7 within abdominal segments 3, 4, and 5. Potentials were recorded with an AxoClamp 2B (Molecular Devices) using custom DAQ routines composed in Matlab (Mathworks). Synaptic potentials were elicited by stimulating segmental motor neurons via a glass suction electrode, a Grass S88 stimulator, and a stimulus isolation unit (Grass Technologies, West Warwick, RI). Single impulses were generated at 0.2 Hz, 330 μs pulse duration, and ~120% of the voltage needed to attain maximal compound EJP amplitude.

### Statistical analyses

All statistical analyses were performed in GraphPad Prism software. Data were assessed for normality using the D'Agostino & Pearson test, and data that fit a normal distribution were analyzed with parametric measures, while data that did not fit a normal distribution were analyzed with non-parametric measures. The statistical analyses utilized are demarcated in the figure legend, with error bars representing SEM.

## Results

**Fig 1:**
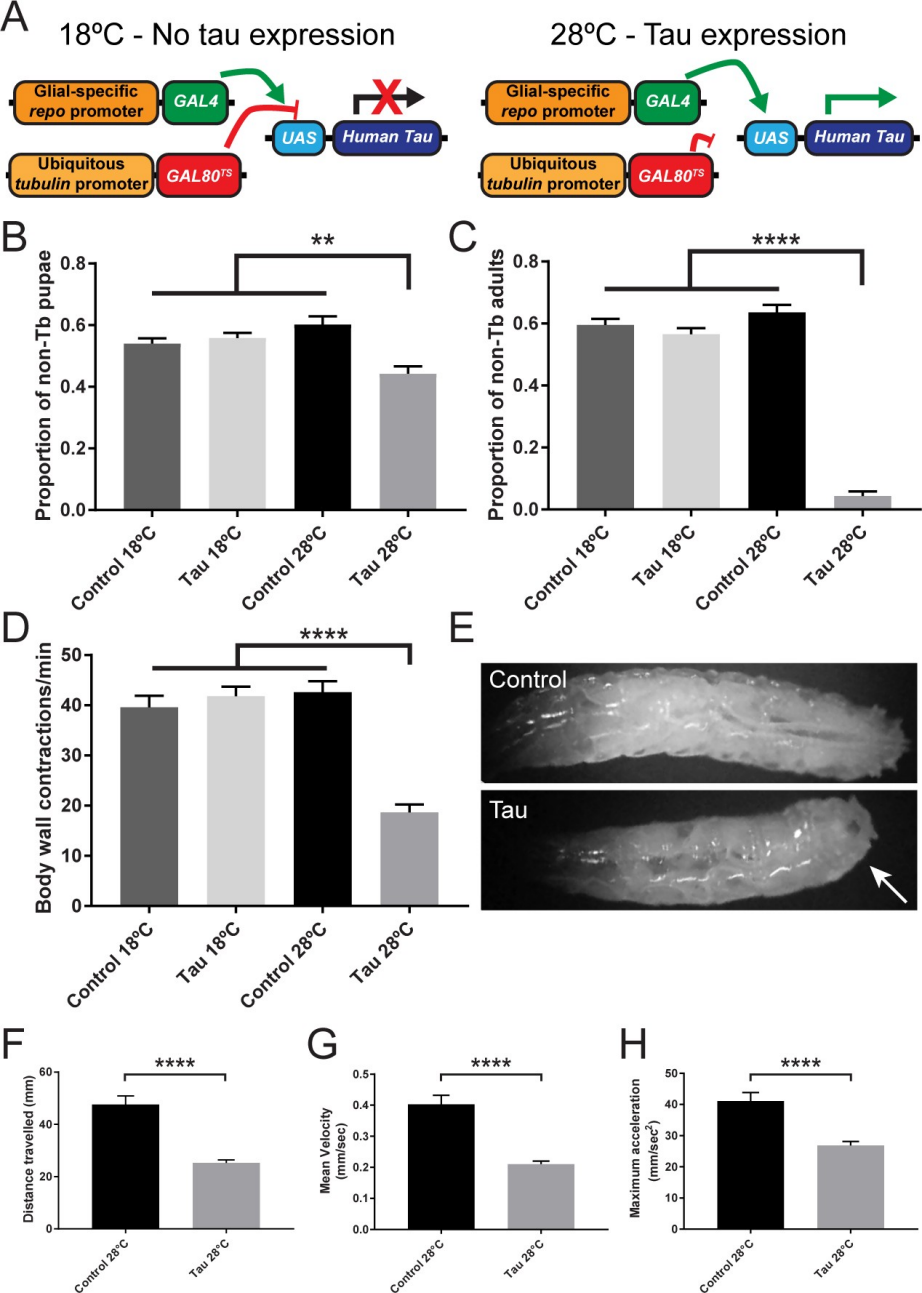
Glial tau expression during development is lethal and causes larval motor deficits. (A) Schematic depicting temperature-sensitive control of GAL4/UAS-driven tau expression by ubiquitous expression of a temperature-sensitive allele of GAL80 (*GAL80*^*TS*^). At 18°C, glial-specific, GAL4-mediated tau expression is repressed by GAL80^TS^, while at 28°C, GAL80^TS^ is unable to repress GAL4 and glial-specific tau expression is achieved. (B) Proportion of pupae that lack the *TM6B*,*Tb* balancer (non-Tb) from control and glial tau transgenic crosses at 18°C and 28°C. (n = 3–4 crosses; 347–916 pupae; ** p < .01, **** p<0.0001, Kruskal-Wallis with Dunn’s multiple comparison). (C) Proportion of eclosed adults lacking the *TM6B*,*Tb* balancer from control and glial tau transgenic crosses at 18°C and 28°C. (n = 3–4 crosses; 205–628 adults; **** p<0.0001, Kruskal-Wallis with Dunn’s multiple comparison). (D) Body wall contractions of control and glial tau transgenic larvae from crosses performed at 18°C and 28°C. (n = 20–30; **** p<0.0001, One-way ANOVA with Tukey’s multiple comparison). (E) Representative light microscopic image of normal control larva and glial tau transgenic larva with dystonic, curled posterior (arrow). (F-H) Larval motility parameters were analyzed with Ethovision during 2 minute trials. (F) Total distance travelled, (G) mean velocity, and (H) maximum acceleration were analyzed from control and glial tau transgenic larvae from crosses performed at 28°C. (n = 20; **** p<0.0001, Mann-Whitney) Error bars represent SEM.

To examine the pathological consequences of developmental human tau expression in *Drosophila* glia, we utilized the GAL4/UAS expression system[30], coupled with a temperature-sensitive allele of the GAL4 repressor, GAL80 (*GAL80*^*TS*^)[24] to achieve glial-specific, temperature-sensitive expression of human tau (Fig 1A)[23]. Both control (*repo-GAL4*,*tub-GAL80*^*TS*^*/TM6B*,*Tb)* and glial tau transgenic (*repo-GAL4*,*tub-GAL80*^*TS*^,*UAS-Tau*^*WT*^*/TM6B*,*Tb*) flies were mated to isogenic *w*^*1118*^ flies to eliminate the *TM6B*,*Tb* balancer chromosome, and these crosses were allowed to developmentally progress through embryonic, larval, and pupal stages to assess organismal development. Crosses performed at 18°C, where tau expression is repressed by *GAL80*^*TS*^, generated normal proportions of pupae and eclosed adult flies in both control and tau transgenic crosses (Fig 1B and 1C). However, while control crosses performed at 28°C generated normal proportions of pupae and eclosed adult flies, glial tau transgenic crosses where tau is expressed in glial cells displayed a reduction in the proportion of tau transgenic pupae (Fig 1B) and eclosed adults (Fig 1C), indicating organismal lethality. These data demonstrate that tau overexpression in glia is toxic during fly development and suggest that the inability of tau transgenic flies to eclose from the pupal casing is the result of a motor phenotype. To test this hypothesis and better characterize the deleterious effects of glial tau expression during development, we performed a crawling assay on control and glial tau transgenic larvae at both 18°C and 28°C. At 18°C, both control and tau transgenic larvae exhibited normal locomotive behavior, however, at 28°C glial tau transgenic larvae displayed impaired body wall contraction behavior compared to control larvae (Fig 1D). These contraction deficits were infrequently accompanied by the curling of the posterior end (Fig 1E), consistent with dystonic paralysis (observed in 2/30 glial tau transgenic larvae, and not observed in 30 control larvae)[31,32]. To better define the locomotor deficits of glial tau transgenic larvae, we analyzed additional motility measures in control and glial tau transgenic larvae crossed at 28°C. Glial tau transgenic larvae displayed significant reduction in distance travelled (Fig 1F), mean velocity (Fig 1G), and maximum acceleration (Fig 1H), further characterizing the motor phenotype associated with larval glial tau expression.

**Fig 2:**
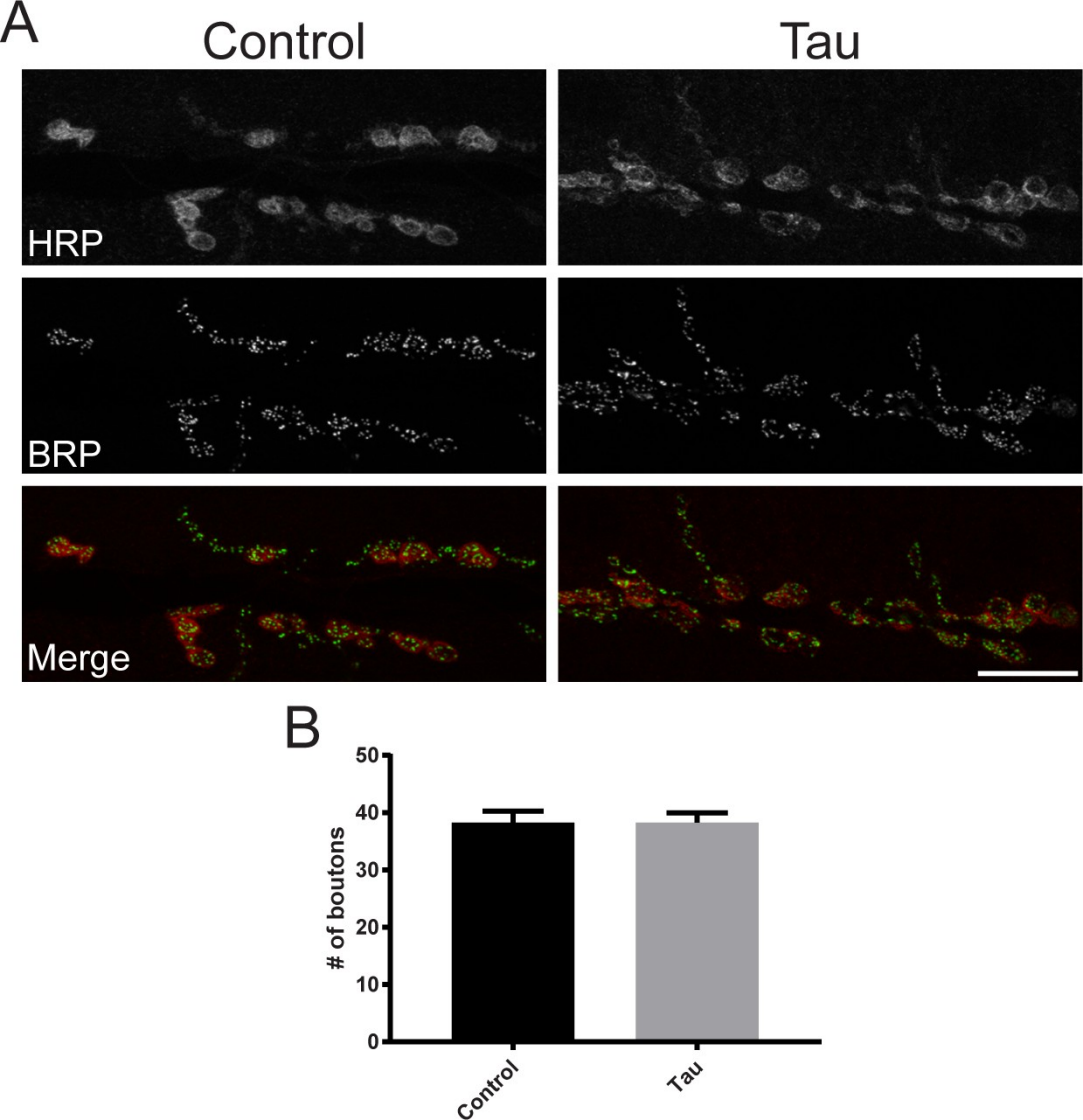
NMJ synapse formation is normal in glial tau transgenic larval. (A) Representative maximum projection confocal images of control and glial tau transgenic NMJ boutons stained with antibodies against HRP (red) and Bruchpilot (BRP, green). (B) Quantification of bouton number at muscle 6/7. (n = 81–101, p = 0.95, Mann-Whitney) Scale bar = 10 μm. Crosses were performed at 28°C.

**Fig 3:**
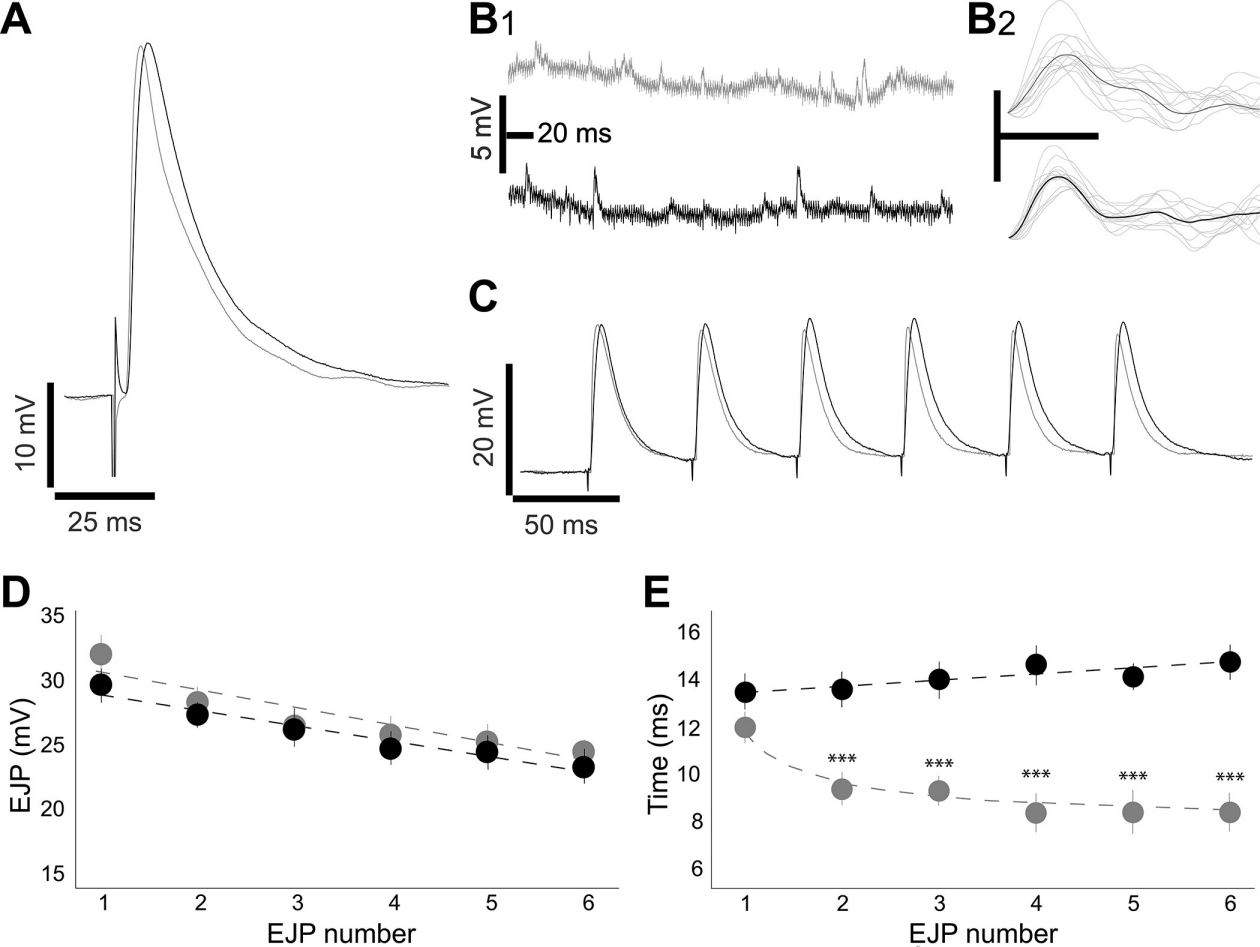
Evoked and spontaneous synaptic activity is normal, while EJP decay time is reduced upon successive stimulation, in glial tau transgenic larvae. (A) Evoked excitatory junction potential waveform is similar between control (black) and glial tau transgenic (grey) NMJ (n = 89, 69, respectively, p = 0.51 amplitude and p = 0.20 decay time, t-tests). (B) Spontaneous miniature end plate potentials occurred at similar frequencies and amplitude (B1). Mini waveforms (B2) were similar between control and glial tau transgenic larvae (n = 46, 48, respectively, peak amplitude: p = 0.42, t-test). (C) An example muscle recording upon presentation of brief (300 ms), high frequency (20 Hz) stimulus train to the motoneuron. (D) Peak compound EJP amplitudes were not statistically different in control and glial tau transgenic larvae (p = 0.45). (E) In contrast, EJP decay time decreased with stimulus number in glial tau transgenic larvae (e.g. stimulus 6 vs. stimulus 1, ***p<0.001, Friedman’s ANOVA), while EJP decay time was unaffected in control larvae across stimuli (p>0.05, ANOVA). EJP decay time was thusly significantly reduced in glial tau transgenic larvae, compared to control (p<0.0001, Friedman’s ANOVA). Crosses were performed at 28°C.

Given that glial tau expression leads to locomotor deficits at 28°C, we tested whether these motor deficits were a result of disruptions at the NMJ. We first imaged and quantified the number of motor neuron boutons on muscle 6/7 in control and tau transgenic larvae crossed at 28°C, and found that glial tau expression did not alter bouton morphology or number (Fig 2A and 2B). Then we determined whether synaptic activity was altered in glial tau transgenic larvae by performing electrophysiological recordings of evoked excitatory junction potentials (EJP) and spontaneous miniature end plate potentials. Single compound EJP recordings from glial tau transgenic larval muscle were electrophysiologically normal compared to control EJPs. Evoked EJP amplitude and decay constants (Fig 3A: Control_ΔV_: 32.01 +/- 2.23 mV, Tau_ΔV_: 29.57 +/- 2.57 mV; Control decay_τ_: 14.07 +/- 1.41 ms, Tau decay_τ_: 11.57 +/- 0.92 ms) and mini frequency and amplitude (Fig 3B; Control freq.: 9.8 +/- 2.19 Hz; Tau freq.: 6.48 +/- 1.96 Hz; Control_ΔV_: 4.2 +/- 0.91 mV, Tau_ΔV_: 3.9 +/- 1.1 mV) were not statistically significantly different in glial tau transgenic larvae. In addition, we utilized brief trains of motoneuronal stimulation at 20 Hz to further evaluate synaptic transmission (Fig 3C). Upon repeated activation of the synapse, EJP amplitude in the muscle of glial tau transgenic larvae was not statistically different from controls (Fig 3D). The decay time of compound EJPs, however, was progressively and significantly reduced in glial tau transgenic larvae compared to controls (Fig 3E) Together with the structural synapse data (Fig 2), these data suggest that while the glial transgenic larval NMJ synapse develops and functions normally during evoked and spontaneous activity, successive stimulation of the motoneuron reveals a reduction in EJP decay time in glial transgenic larvae.

**Fig 4:**
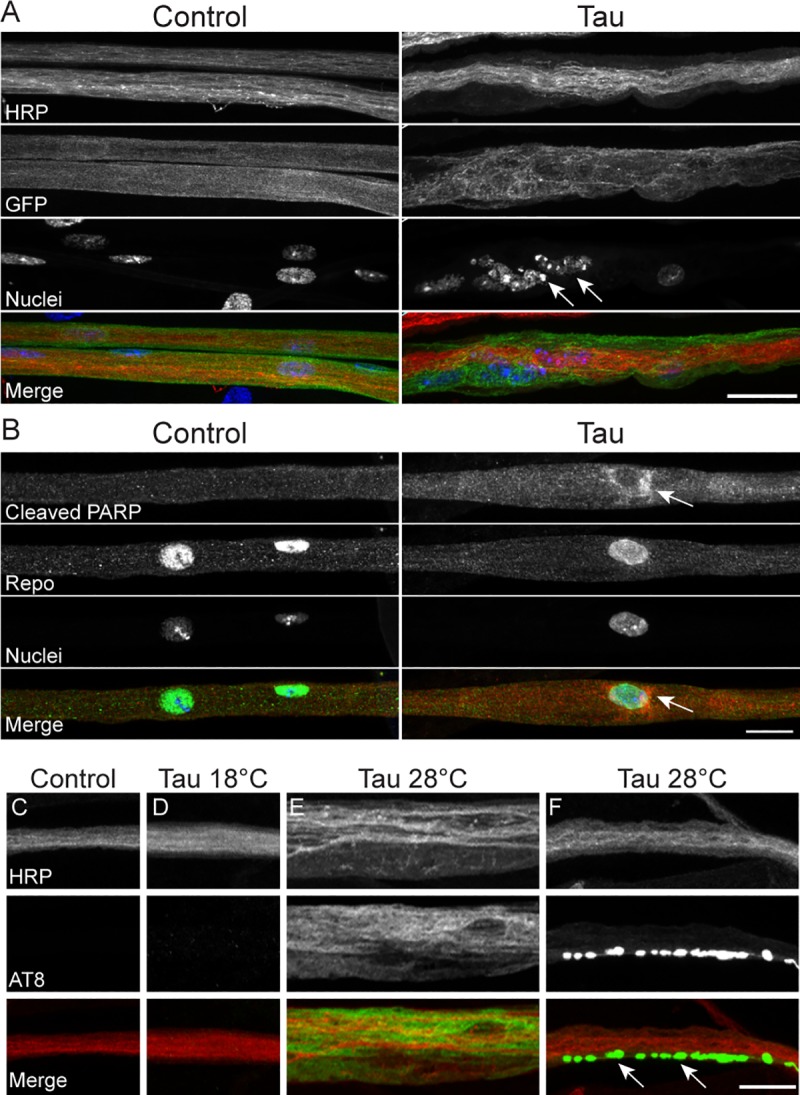
Larval glial tau expression disrupts axonal and glial morphology, induces glial cell death, and yields hyperphosphorylated tau species. (A-C) Representative maximum projection confocal images of control and glial tau transgenic larval segmental nerves. (A) Co-expression of membrane-bound GFP reveals normal axonal integrity and glial ensheathment in control larval segmental nerves (left) and abnormal nerve defasciculation, glial membrane disruption and fragmented glial nuclei (arrows) in glial tau transgenic nerves (right). Nerves were stained using antibodies against HRP (red, axons), GFP (green, glia) and Hoechst (blue, nuclei). Crosses performed at 28°C; Scale bar = 10 μm. (B) Co-expression of a cleavable fragment of PARP and subsequent immunofluorescence staining of segmental nerves stained with antibodies against cleaved PARP (red) and the glial-specific protein, repo (green) reveals an absence of caspase activation in control segmental nerves (left), and peri-nuclear caspase activation in glial cells surrounding tau transgenic segmental nerves (arrow, right). Crosses performed at 28°C; Scale bar = 10 μm. (C-F) Larval segmental nerves stained with antibodies against HRP (red, axons) and AT8 (green) do not display AT8 staining in (C) control nerves or (D) glial tau transgenic nerves crossed at 18°C, while at 28°C, glial cells that wrap tau transgenic nerves display both (E) diffuse and (F) aggregate-like tau species (arrows). Scale bar = 5 μm.

Next, axonal and glial perturbations of the segmental nerve were assessed by immunofluorescence staining of the glial-wrapped PNS nerves to determine whether glial tau expression alters axonal morphology. Larval segmental nerves are comprised of motor and sensory neurons, and are wrapped by glial membranes[21,22], similar to non-myelinating Schwann cells in the vertebrate PNS. Glial membranes were visualized by glial-specific expression of a membrane-bound GFP construct and neuronal axons were visualized with an antibody against HRP, which specifically recognizes *Drosophila* axons[33]. Immunofluorescence analysis of cellular morphology revealed glial tau expression induces disorganized glial membrane wrapping, nerve defasciculation, and fragmented glial nuclei (Fig 4A). Nuclear fragmentation is an indicator of cell death, and given that tau induces apoptotic cell death when expressed in adult glial cells[23], we assessed whether tau expression was similarly toxic to larval glial cells. To determine if glial tau expression induced apoptotic cell death, we co-expressed a fragment of the human caspase-cleavable PARP protein in glial cells using the *UAS-CD8-PARP1-Venus* construct[34]. The presence of cleaved PARP is used as an indicator of caspase activation, consistent with apoptotic cell death[35,36]. Immunofluorescence using antibodies specific for cleaved PARP and the glial-specific protein, repo, revealed that glial tau transgenic larvae contain nerve-associated glial cells that display peri-nuclear cleaved PARP, while control larvae do not (Fig 4B), demonstrating that larval glial tau expression is toxic to axon-associated glial cells of the PNS.

The accumulation of hyperphosphorylated tau pathology in glial cells is a prominent feature of several tauopathies[2,37], and hyperphosphorylated tau tangles are formed when human tau is expressed in adult *Drosophila* glial cells [23]. To determine if similar tauopathy-associated phosphorylated tau species are generated when tau is expressed in larval glial cells, we performed immunofluorescence staining using the phospho-specific antibody, AT8, which recognizes phosphorylation events on human tau at residues Ser^202^ and Thr^205^. We found that these phosphorylation events were absent from control glial cells (Fig 4C) and tau transgenic glial cells crossed at 18°C (Fig 4D), but were present when tau was expressed in glial cells at 28°C (Fig 4E and 4F). Phosphorylated tau largely existed as diffuse staining (Fig 4E), though aggregate-like species of phosphorylated tau were observed (arrows, Fig 4F). Taken together, these data show that larval glial tau expression induces toxicity in the larval PNS, and the presence of disease-associated phosphorylation events on tau demonstrates that larval glial kinases have the capacity to phosphorylate human tau, similar to adult *Drosophila* glial kinases[23].

**Fig 5:**
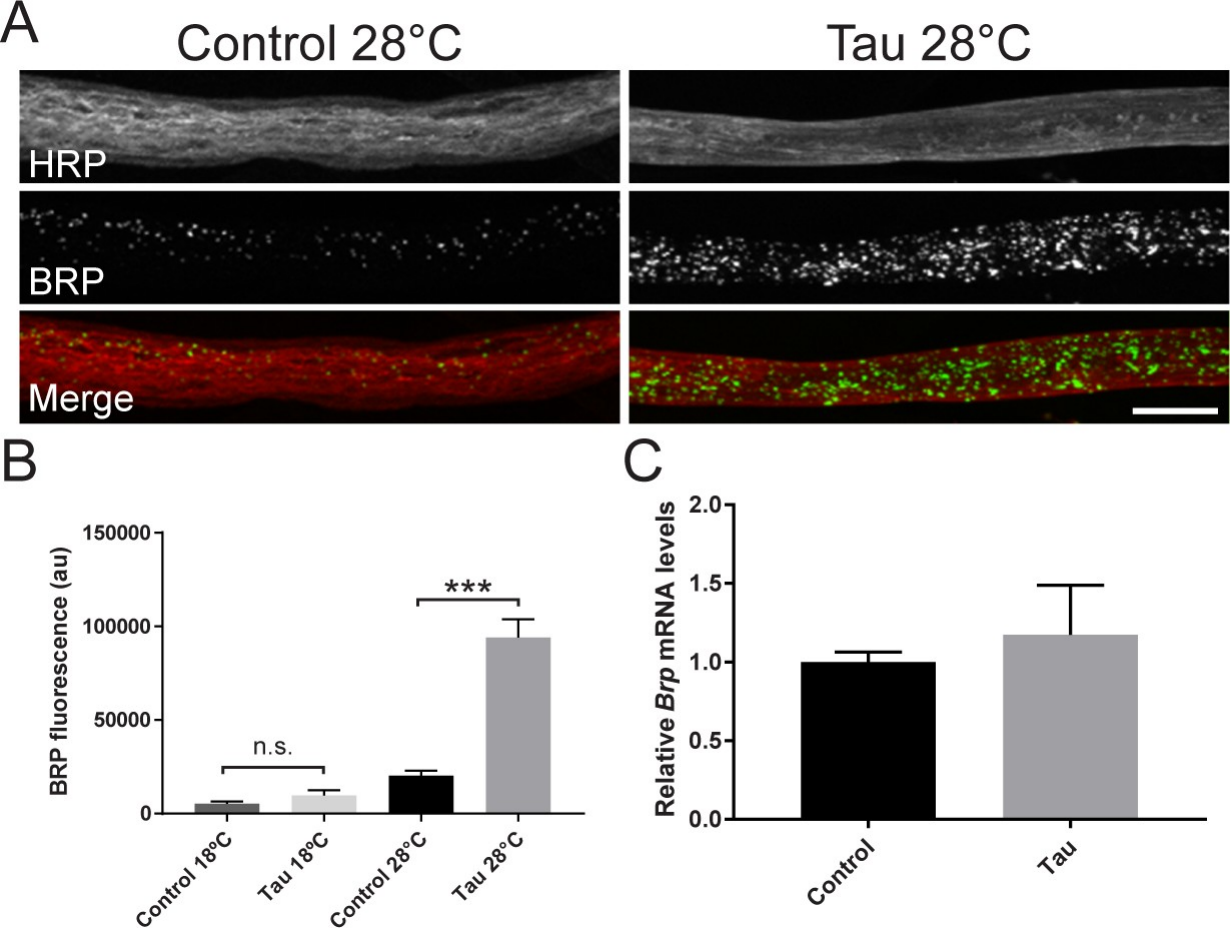
The presynaptic protein, Bruchpilot, accumulates in the axons of glial tau transgenic segmental nerves. (A) Representative maximum projection confocal images of control and glial tau transgenic larval nerves crossed at 28°C and stained with antibodies against HRP (axons, red) and the presynaptic protein, Bruchpilot (BRP, green). Scale bar = 5 μm (B) Quantification of BRP fluorescence in control and glial tau transgenic larvae crossed at 18°C (n = 29–38, p = 0.43, Mann Whitney) and 28°C (n = 73–75; ***p<0.001, Mann-Whitney; Error bars represent SEM). (C) Relative *Brp* mRNA levels in control and glial tau transgenic larvae crossed at 28°C. (n = 5; p = 0.69, Mann-Whitney). Error bars represent SEM.

Disruption of axon-associated glial cells has the capacity to deleteriously affect associated axons, underscoring the necessity of maintaining proper axonal-glial interactions[38,39]. Furthermore, murine tauopathy models have previously shown that glial-specific human tau expression induces neuronal axonal abnormalities and trafficking deficits[6,7,15]. To determine if similar deficits accompany the glial tau-mediated morphological disruption of PNS segmental nerves, we investigated the trafficking of a principle component of the presynaptic NMJ active zone, Bruchpilot (BRP)[40]. We observed a significant accumulation of BRP in the axons of glial tau transgenic segmental nerves, compared to control when crossed at 28°C, but not at 18°C (Fig 5A and 5B). The increased BRP levels did not reflect elevated *Brp* transcription, as relative *Brp* mRNA levels were similar between control and tau crossed at 28°C (Fig 5C), suggesting that there is a disruption in BRP trafficking and/or degradation in the axons of glial tau transgenic nerves.

## Discussion

This study aimed to assess the developmental consequences of human tau expression in *Drosophila* glial cells on PNS development and function. We show that glial tau expression interrupts the developmental progression of the fruit fly by stalling development at the pupal stage. Our analysis of the PNS at the larval stage revealed that glial tau expression results in larval motor deficits, apoptotic glial cell death and the formation of tauopathy-associated phosphorylated tau species. These glial perturbations were associated with morphological disruptions to segmental nerve axonal integrity and induced the aberrant axonal accumulation of the presynaptic protein, BRP. The non-cell-autonomous neuronal effects of glial tau expression, however, did not alter synapse structure at the NMJ, suggesting that glial tau toxicity manifests after initial PNS synapse formation is complete.

Tauopathies are a class of neurodegenerative disorders characterized by the accumulation of hyperphosphorylated tau in neuronal and glial cells of the CNS[2,3,41]. Though these tau lesions are primarily localized to the CNS[9,42–44], tau accumulations within the PNS have been described. The pathological significance of these lesions remains unclear[45], and even less clear is whether glial cells of the PNS display tau pathology, and are affected in these disorders. Within the CNS, tau pathology spreads from neuronal to glial cells[46,47], and given that tau appears to spread to PNS neurons[9], it raises the possibility that tau pathology could spread to PNS glial cells. It is therefore important to understand the consequences of PNS glial tau pathology from a basic biological perspective, but also in relation to tauopathy pathogenesis. Using the *Drosophila* model system, we have found that glial tau expression is lethal to the developing fly, and disrupts the integrity of glial-wrapped PNS segmental nerves, as the nerves become defasciculated and glial membranes are deformed. The deleterious effects of glial tau expression are in line with murine tauopathy studies that have highlighted tau-mediated PNS disruptions. To date, there are no mouse tauopathy models specifically expressing tau in neuronal or glial cells of the PNS, but mice expressing tau in all cells of the nervous system, including PNS Schwann cells, revealed that tau expression induces degeneration of myelin from the Schwann cells that ensheath the sciatic nerve[15], consistent with the data presented here. Peripheral nerve degeneration and associated myelin abnormalities are also a feature of the PS19 tau transgenic mouse model, where tau is also expressed in the PNS and CNS[14]. Together, these studies highlight a pathogenic role for tau when introduced into the PNS, and in particular, emphasize the potential for glial tau to disrupt peripheral glial-axonal integrity.

Previous studies in *Drosophila* tauopathy models have successfully elucidated mechanisms of tau-mediated cell death[48], and while most of these models express tau in the adult *Drosophila* CNS to uncover age-related degenerative mechanisms, studies expressing tau in the developing nervous system have also yielded valuable insights into tau-mediated toxicity. Our findings that tau induces larval PNS glial cell death is similar to the toxic effects of glial tau expression in adult *Drosophila* CNS glial cells[23], and demonstrates that glial tau induces cell death in both contexts. Other studies examining tau toxicity during *Drosophila* development have consisted of targeted tau expression to larval motor neurons in the PNS, and have shown that while human tau expression in larval neurons disrupts synapse formation and physiology[49,50], these deleterious developmental effects of neuronal tau expression occur independent of neuronal cell death[49]. This is in contrast to adult neuronal tau expression models, which are characterized by extensive neuronal death[51,52]. These disparate findings highlight potential mechanistic differences of neuronal tau toxicity between developmental and adult and/or between CNS and PNS contexts. However, tau phosphorylation is an important determinant of tau toxicity in both neuronal contexts[53,54], underscoring important similarities between these models. Similarly, neuronal tauopathy models have demonstrated that different tau isoforms yield differential toxicity during development and in the adult. For example, 0N3R tau is particularly toxic to larval neurons [55], while 0N4R more readily promotes neurodegeneration and impairs learning and memory in the adult [55,56], perhaps the result of tau isoform-specific effects on synaptic transmission at adult CNS synapses[57]. Here, we characterize the pathological effects of glial expression of 0N4R tau, but given the isoform-specific effects of tau on neuronal physiology, an interrogation into tau isoform effects on glial cells is warranted.

We find that developmental glial tau expression yields the formation of aggregated, phosphorylated tau species indicating that glial kinases that phosphorylate human tau are active at the larval stage. However, given that insoluble, fibrillary tau tangles are generated after 20 days of tau expression in adult glia, these larval glial tau aggregates may represent early pre-tangle structures [58]. Additional experiments examining the solubility and ultrastructural properties of these larval glial tau accumulations are therefore necessary to determine how similar these structures are to the tangles that are generated in the adult *Drosophila* glial tauopathy model [23].

The apoptotic cell death of larval PNS glial cells is similar to glial tau-mediated apoptotic cell death of adult CNS glial cells. In the adult model, glial cell death was controlled by tau-mediated disruption of JAK/STAT signaling[23], a pleiotropic signaling pathway critical for glial cell function[59,60], and it will therefore be important to assess whether similar disruption in JAK/STAT signaling is responsible for developmental glial tau toxicity in the PNS. The rapid time course of tau-mediated toxicity in this developmental tauopathy model will expedite these studies aimed at the mechanistic dissection of glial tau toxicity.

Neuronal-glial interactions are essential for the early development of the *Drosophila* PNS[38]. We found that glial tau expression induces larval locomotor deficits and organismal lethality at the pupal stage, without affecting larval NMJ structural synapse formation. These data suggest that tau begins to exert toxicity during the larval stage, but does not affect initial NMJ patterning. *Drosophila* PNS development begins during embryonic stages, where glial cells migrate along segmental nerve axons and wrap them[20]. Targeted ablation of peripheral glial cells results in aberrant neuronal axonal pathfinding and disrupted axogenesis[38,61], highlighting the essential nature of these glial interactions. The driver used in this study, *repo-GAL4*, turns on expression during embryonic stages[62], and while a detailed analysis of embryonic development was not performed, we can infer from the lack of a structural phenotype at larval stages that the expression of tau does not interfere with the glial-dependent embryonic patterning of the *Drosophila* NMJ[38]. This suggests that either glial tau expression is not toxic to embryonic glial cells or that the toxic effects of glial tau expression take time to be uncovered.

In addition to their role in development, glial cells maintain healthy axons through a combination of contact-mediated and signaling pathways[63,64]. Our finding that glial tau expression impairs locomotor function in the absence of a structural NMJ synaptic phenotype, suggests that the developmental consequences of glial tau toxicity may impair peripheral neuronal function after synapse formation is complete. Whereas postsynaptic amplitude is unaffected in glial tau transgenic larvae, a significant decrease in the duration of synaptic potentials upon repeated stimulation of the motoneuron was observed. The mechanism underlying these effects is unclear, however, perturbation of the glial sheath surrounding the NMJ may contribute to this effect[21]. These potential contact-mediated effects may also occur in the context of altered glial signaling. Previous data suggests that prodegenerative, glial-derived signaling exists in the *Drosophila* neuromuscular system, where the secreted factor, *Drosophila* TNF-alpha (eiger) is expressed in a subset of peripheral glia and its receptor (Wengen) is expressed in motoneurons[63]. Conversely, glial cells also secrete neurotrophic factors that support neuronal integrity[65]. Thus, glial tau expression may alter the secretion of these glial factors, by either increasing prodegenerative factors or decreasing trophic factors, or a combination thereof, which may serve as a precipitating factor in deleteriously affecting neuronal function.

We found that glial tau transgenic larval segmental nerves are characterized by axonal accumulations of the pre-synaptic protein, BRP. This accumulation occurs in the context of normal *Brp* transcription and normal synaptic BRP localization, which may reflect a deficiency in BRP trafficking and/or degradation. Similarly, murine glial tauopathy models display motor deficits, concomitant with axonal trafficking defects, suggesting a glial tau-dependent mechanism of neuronal dysfunction[7,14]. While this mechanism remains unclear, other studies of *Drosophila* larval segmental nerves suggest that neuronal kinase dysregulation is important in regulating BRP trafficking. For example, neuronal overexpression of Par-1 kinase, a kinase that phosphorylates tau and modulates its affinity for microtubules, results in BRP accumulation[66,67]. This BRP accumulation, however, appears to be independent of Par-1’s role in phosphorylating tau, as Par-1-mediated BRP accumulation is not observed in *tau* mutant larvae[66]. Similarly, dysregulation of the kinases, serine protein kinase (SRPK79D) and casein kinase 2 (CK2α), are also characterized by aberrant BRP accumulation in larval axons[27,68]. These studies underscore the importance of kinase activity regulation in maintaining normal axonal trafficking of key pre-synaptic active zone proteins, and additional experiments will be necessary to ascertain if glial tau expression alters neuronal kinase activity. Given that dysregulated kinase activity, particularly with tau as the substrate, is a defining feature of tauopathies[69], these experiments will assist in better defining the role of disrupted neuronal-glial interactions in tauopathy pathogenesis. However, it should be noted that using the pan-glial driver, *repo-GAL4*, our studies are limited in that they do not distinguish between glial cell subtypes in mediating these neuronal deficits. *Drosophila* segmental nerves contain several specialized glial cells (e.g. wrapping, subperineurial and perineurial glial cells) that are necessary for proper ensheathment and function[20,70]. Future experiments using glial cell-type specific GAL4 drivers in this larval glial tauopathy model will be necessary in teasing apart these tau-mediated cell type-specific effects on neuronal structure and function.

In summary, we have generated a larval *Drosophila* model of glial tauopathy that results in larval locomotor deficits, pupal lethality, morphological disruption of PNS segmental nerves, and aberrant axonal accumulation of the pre-synaptic protein, BRP. Together, these results highlight a new model system in which to investigate the mechanisms underlying the deleterious effects of glial tau expression on neuronal-glial interactions in the PNS, and provide additional evidence in support of a pathogenic role for glial tau pathology in tauopathy pathogenesis.
